# Co-Expression of Pig IL-2 and Fusion Bovine Cathelicidin Gene by Recombinant Plasmids in Yeast and Their Promotion of Mouse Antibacterial Defense

**DOI:** 10.3390/biology11101491

**Published:** 2022-10-12

**Authors:** Jianlin Chen, Junjie Peng, Changjun Ma, Linhan Zhang, Xueyin Wu, Hong Wei, Jianglin Li, Xuebin Lü, Rong Gao

**Affiliations:** 1 School of Laboratory Medicine, Collaborative Innovation Center of Sichuan for Elderly Care and Health, Chengdu Medical College, Chengdu 610500, China; 2 Key Laboratory of Bio-Resource and Eco-Environment of Ministry of Education, College of Life Sciences, Sichuan University, Chengdu 610065, China; 3 National Engineering Research Center for Biomaterials, Sichuan University, Chengdu 610065, China; 4 Institute of Precision Medicine, The First Affiliated Hospital, Sun Yat-sen University, Guangzhou 510080, China; 5 Sichuan Academy of Animal Science, Chengdu 610066, China

**Keywords:** IL-2 gene, bovine cathelicidin genes, co-expression, promotion, mouse immunity, recombinant Pichia pastoris

## Abstract

**Simple Summary:**

1. Oral inoculation with recombinant yeast co-expressing pig IL-2 and fused bovine cathelicidin (FBC) significantly enhanced systemic immunity in mice. 2. The inoculated mice displayed stronger resistance against *E. coli* or *S. aureus* infection. 3. Recombinant yeast co-expressing PIL-2 and FBC could facilitate the development of new immunopotentiator to prevent the infection of antibiotic-resistant bacteria.

**Abstract:**

In order to develop an effective and safe immunomodulator to enhance the antimicrobial bioactivity and immunity of animals against infectious bacterial diseases, a recombinant plasmid pGAPZαA-IL2-B co-expressing pig interleukin-2 (PIL-2) and fused bovine cathelicidin (FBC) genes were constructed using the 2A self-cleavage technique. After being expressed in Pichia pastoris strain SMD1168, the recombinant yeast was administered orally to 5-week-old female ICR mice. The control mice were similarly dosed with P. pastoris with a blank plasmid or FBC recombinant plasmid alone. At 28 days post-treatment, the mice were challenged intraperitoneally with virulent strains of either *E. coli* or *S. aureus*. Compared with the control groups, the mice that received recombinant yeast co-expressing PIL-2/FBC manifested significant increases in the number of leukocytes, CD4+ and CD8+ T cells, IgG, and the gene expressions of TLRs(TLR1,4,6,9), antimicrobial peptides(CRP4 and CRAMP) and cytokines (IL-2, 4, 6, 7, 12, 15, 23, IFN-γ, and TNF-α) in the blood. Furthermore, the treated mice displayed significantly higher survival than the other two control groups after the challenge. These results suggest that the antimicrobial activity and immunity of animals can be effectively enhanced by the in vivo co-expression of IL-2 and the FBS gene, which can facilitate the development of new immunopotentiation molecules to overcome the infection of antibiotic-resistant bacteria.

## 1. Introduction

Increasing cases of antimicrobial resistance (AMR) and the re-emergence of infectious diseases require the urgent development of new and effective antimicrobial strategies [1,2]. Natural antimicrobial peptides (AMPs), which have been identified in numerous organisms, have attracted attention due to their activity as membrane pore formers and their effectiveness against antibiotic-resistant bacteria [3]. Recent studies have also shown that AMPs exhibit a wide range of functions, ranging from direct antimicrobial properties to immunomodulatory effects [4,5].

In addition to their well-recognized direct antimicrobial activities, cathelicidins, a major group of AMPs found in many mammalian species, can stimulate a diverse range of biological effects involved in initiating and amplifying host innate and adaptive immune responses [6,7]. Innate defense regulator (IDR) peptide IDR1002, a linear derivative of cathelicidins, provokes the induction of anti-inflammatory cytokines to limit excessive inflammation and control bacterial infections [8]. Indolicidin, a derivative of bactenecin, has the collective ability to inhibit LPS-induced proinflammatory cytokines, induce chemokines, and block endotoxin activity [9]. Bac2A, a 12-amino acid cathelicidin, has very weak antiendotoxic and chemokine-inducing properties but acts directly to induce chemotaxis in THP-1 cells [10]. In addition to their antimicrobial activity, indolicidin and Bac2A have been shown to have diverse and complementary immunomodulatory functions. CP10A, a modified version of indolicidin including a Pro to Ala mutation, retains its activity while increasing its potency against Gram-positive bacteria [11].

Interleukin-2 (IL-2), which is recognized as a major T cell growth factor and enhancer of the proliferation of activated cytotoxic lymphocytes [12], has also been used as an adjuvant to regulate the immunity of animals because of its safety and effectiveness [13]. However, the use of single IDR1002, indolicidin, Bac2A, CP10A, or IL-2 alone as antimicrobial drugs is usually insufficient to achieve strong and persistent immunity in animals because of their rapid degradation and low bioactivity in vivo; therefore there is an urgent need to develop novel safe and effective immunopotentiator to improve animal resistance against various infectious diseases.

Some strategies exist to solve the problems of the poor bioactivity and short half-time in vivo. Recently, the fusion of multivalent AMPs genes has been reported to produce stronger bioactivity than the AMP monomer genes themselves [14]. Additionally, the use of fused AMPs and cytokines would obviate the adverse effects of multiple injections necessary for the induction of an adequate immune response by single cytokines or AMPs.

The yeast expression system is generally regarded as a safe organism and has the advantages of both prokaryotes (high expression level, ease of scale-up, and low cost) and eukaryotes (post-translational modifications) while avoiding the problems of endotoxins and the inherent genetic safety in the use of bacterial and viral vectors [15]. In addition, yeast has been used as a probiotic for the regulation of intestinal microbial homeostasis and the modulation of immune responses as an immuno-stimulant. Administered orally, it resists degradation in the gastrointestinal tract, with a high survival rate in the stomach and small intestine [16,17], meaning that it is an effective bioencapsulation vehicle for the protection of recombinant molecules.

While many studies have focused on using IDR1002, indolicidin and Bac2A, CP10A, and IL-2 singly for their antimicrobial and immunomodulatory activities, recombinants expressing fused bovine cathelicidins (FBC) and IL-2 have received less attention. Here, we describe the construction of a recombinant yeast co-expressing pig IL-2 and FBC linked using the 2A self-cleavage technique and its effect on the immune response and antimicrobial activity in vitro and in vivo.

## 2. Materials and Methods

### 2.1. Construction of Recombinant Plasmid

Firstly, the cDNAs of pig IL-2 and fused bovine cathelicidins (FBC) containing indolicidin, IDR1002, Bac2A, and CP10A were, respectively, subcloned from recombinant plasmids VPIL2 [13] and VAB (FBC was inserted into plasmid PVAX1, the recombinant gene FBC(TAP-Bac2A-linker-IDR1002-linker-indolicidin-linker-CP10A-His-tag) was constructed in our laboratory by splicing overlap extension PCR (SOE PCR) [18]. The tissue plasminogen activator (TAP) signal sequence and these four cathelicidin genes were connected with each other by the GSGDDDDK linker. The FMDV 2A self-cleavage peptide and downstream α-factor signal sequence for secretion were used to generate a multicistronic cassette linking the pig IL-2 and FBC genes. Then, to construct a fusion gene of pig IL-2, 2A-α, factor and FBC (PIL-2/2A-α/FBC), six oligodeoxynucleotide fragments were designed and synthesized. The fused gene PIL-2/2A-α/FBC was prepared by SOE PCR [18]. The stop codon of pig IL-2 and the start codon of FBC were deleted, and the EcoRI and Xbal restriction sites were added to the ends. The fused PIL-2/2A-α/FBC gene was digested with EcoRI and Xbal and then inserted into the *E. coli*–Pichia pastoris shuttle plasmid pGAPZαA (Invitrogen, Carlsbad, CA, USA), a eukaryotic expression vector containing the GAP promoter, α-factor signal sequence for secretion and a polyhistidine tag (6× Histidine) for fusion protein expression. Separately, the FBC gene with added EcoRI and Xbal restriction sites and employing primers FBC-F’ and FBC-R’ was also cloned into pGAPZαA. All of the primers were designed by us and synthesized by Sangon Biotech Co., Ltd., Shanghai, China (Table 1). The recombinant plasmids containing PIL-2/2A-α/FBC and FBC were verified using double restriction enzyme digestion and sequencing, which were, respectively, designated as pGAPZαA-PIL2-FBC and pGAPZαA-FBC. The flow chart of the recombinant plasmid construction is shown in Supplementary Materials Figure S1.

### 2.2. Yeast Transformation

The plasmids pGAPZαA-PIL2-FBC, pGAPZαA-FBC, and pGAPZαA were linearized with AVRII and expressed in Pichia pastoris strain SMD1168 by electroporation. Ten µg aliquots of each plasmid were gently mixed with 100 µL electrocompetent Pichia pastoris SMD1168 in a pre-cooled 0.2 cm electroporation cuvette (Bio-Rad, Hercules, CA, USA), incubated on ice for 5 min, then pulsed at 1500 V and 200 Ω for 5 min. Following the addition of 1 mL of ice-cold 1.0 M sorbitol, the cuvette contents were transferred to a 1.5 mL microcentrifuge tube and incubated at 28 °C for 2 h. The cells were then plated on a YPD medium (1% yeast extract, 2% peptone, 2% D-glucose) in 2% agarose containing 100 µg/mL zeocin and incubated at 28 °C for 3 days. The correct genomic integration of the FBC and PIL-2/FBC genes was confirmed using zeocin selection and genomic PCR.

### 2.3. Fusion PIL2/FBC Protein Expressed by Pichia Pastoris

The shuttle plasmid pGAPZαA has an α-factor signal sequence for secretion and a polyhistidine tag for fusion protein expression, which enables fusion proteins to secrete into supernatant and to be labeled with affinity tags, six consecutive histidine residues. Pichia pastoris strain SMD1168 with pGAPZαA-PIL2-FBC, pGAPZαA-FBC, and pGAPZαA were cultured in a YPD medium with shaking at 30 °C for 72 h, and their OD600 were 25. The recombinant yeast ferment supernatants were separated by centrifugation. The expressing amounts of the His-tagged recombinant proteins were identified by using the His-tag detection ELISA kit (Cayman, Ann Arbor, MI, USA). 

### 2.4. Digestion Analysis In Vitro

The *Pichia pastoris* recombinant cells were cultured in a YPD medium with shaking at 30 °C for 72 h, OD_600_ 25. The supernatants of the recombinant yeast fermentation were separated by centrifugation and then filtered through a 0.2 µm pore size. To mimic digestion in the stomach and intestine, in vitro pepsin and the trypsin digestion of the recombinant yeast ferment supernatants were performed by modification of the method of Garrett [19]. Then, 3.8 mL of the recombinant yeast ferment supernatant was pre-cooled on ice prior to acidification (pH 2) with 1 mol/L HCL and the addition of 0.2 mL pepsin (10 mg/mL) at 37 °C for 1 h to mimic stomach digestion. The pH of the recombinant yeast ferment supernatant (3.8 mL) was adjusted to 7.0 by adding 2 mol/L of sodium bicarbonate followed, by the addition of 0.2 mL of trypsin (10 mg/mL) at 37 °C for 1 h to simulate intestinal fluid. All kinds of digested recombinant yeast ferment supernatants were adjusted back to the original pH of yeast ferment supernatant before they were used for lymphocyte proliferation and antimicrobial assay in vitro.

### 2.5. Pig Lymphocyte Proliferation Assay

The viable cells were quantitated using the Cell Counting Kit 8 (CCK8; Dojindo Laboratories, Kumamoto, Japan).

The pig blood was collected from Landrace pigs, and the lymphocytes were isolated and cultured with 5 µg/mL Con A at 37 °C, 5% CO_2_ for 24 h, then the pig lymphoblasts were separated by FicollEthics-Hypaque density gradient centrifugation (550 g, 20 min), and then adjusted to 1 × 10^6^ cells/mL in RPMI 1640 complete culture medium, and incubated in triplicate 50 µL aliquots with 50 µL different recombinant yeast supernatants in 96-well culture plates. At 48 h, 10 µL of CCK8 solution (5 mg/mL in PBS) was added to each well for an additional 2 h of incubation at 37 °C, 5% CO2 to permit color development. Absorbance at 450 nm was measured in a microplate reader Model 680 (Bio-Rad, Hercules, CA, USA).

### 2.6. Antimicrobial Assay

The antimicrobial bioactivity of the recombinant Pichia pastoris cells was measured by the inhibition of 4 pathogen strains (standard strains: *E. coli* ATCC25922; *S. aureus* ATCC26112; multi-antibiotic-resistant strains *E. coli* and *S. aureus*, provided by Prof. Hongning Wang in our lab).

The cultures of the pathogen strains (5 × 10^4^ CFU in 100 µL) were added to the wells of 96-well plates, followed by the addition of 100 µL aliquots of the recombinant yeast fermentation supernatants, which were treated with pepsin or trypsin, respectively, for 4 h at 37 °C. Turbidities, as a measure of bacterial growth, were measured in a microplate reader Model 680 (Bio-Rad, Hercules, CA, USA) at 600 nm.

### 2.7. Animal Experiment

Thirty healthy 5-week-old (18–22 g) female ICR mice were purchased from the Animal Experiment Center of Sichuan and were randomly divided into three groups, including the blank control, FBC, and PIL-2+FBC, with 10 mice per group. They were orally fed 0.6 mL of different fresh recombinant yeast cultures (OD_600_ = 25) by intragastric administration using a feeding tube every three days and were reared under the same condition for 5 weeks. The mice in the blank control group were orally administered with the cultures of *Pichia pastoris* strain SMD1168 with empty plasmid pGAPZαA (OD_600_ = 25), whereas the mice in the FBC group and PIL-2+FBC groups were orally fed with the recombinant yeast cultures, respectively, with the pGAPZαA-FBC and PIL-2+FBC recombinant with the pGAPZαA-IL2-FBC plasmids and their concentrations were 25 OD_600_. The EDTA-K2 blood samples (about 100 μL) were collected weekly from the tail veins of mice for 5 weeks to analyze the immune cells, immunoglobulin titers, and the expression levels of interleukin genes. On 28 days post-inoculation, all of the mice were intraperitoneally challenged with 0.1 mL of 1 × 10^8^ CFU/mL virulent multi-antibiotic-resistant strains *E. coli* and *S. aureus*. Mice mortality was recorded daily for one-week post-challenge, and the surviving mice were euthanized to observe the possible lesions at 5 weeks post-inoculation.

The mouse experiment was approved by the Animal Ethics Committee (ACE) of the Animal Experiment Center of Sichuan University (SYXK-Chuan-2019-172). The care and use of experimental mice fully complied with Chinese animal welfare laws, guidelines, and regulations.

### 2.8. Immunological Assays

#### 2.8.1. Assay of Immune Cells Quantity in Peripheral Blood

The quantitation of leukocyte cells in the tail venous blood samples collected weekly from each mouse (about 30 μL per group) was performed using a Mindray BC-3000 hematology analyzer.

#### 2.8.2. Measurement of Th and Tc Cells by Flow Cytometry

Anti-mouse CD4 and CD8 mAbs, labeled with peridinin-chlorophyll-a-protein-cyanine 5.5 complex (PerCP-Cy5.5) and phycoerythrin (PE), were purchased from eBioscience (Thermo Fisher, Waltham, MA, USA). Fifty μL of venous mouse blood were mixed with 50 μL of normal saline and incubated in the dark with 1 μL of PerCP-Cy5.5 labeled anti-CD4 and 1 μL of PE-conjugated anti-CD8a for 30 min. One ml (5% *v*/*v*) of lysing solution (Becton Dickinson, Franklin Lakes, NJ, USA) was then added for 5 min to ensure the complete lysis of the erythrocytes, and the surviving cells were washed twice with PBS, with centrifugation at 500 g for 5 min between each step. Finally, the cells were resuspended in 150 μL of PBS and analyzed in a FACScan flow cytometer (Becton Dickinson). 

#### 2.8.3. IgG Determination by Sandwich ELISA

Mouse IgG, IgG1, and IgG2a quantitation ELISA kits, purchased from R&D Systems (R&D Systems Inc., Minneapolis, MN, USA), were employed to measure the levels of IgG, IgG1, and IgG2a in the sera of the venous tail blood collected weekly. Capture antibody-coated 96-well plates were incubated with serially diluted serum samples and standards for 1 h at ambient temperature. HRP-conjugated goat anti-mouse secondary antibodies IgG, IgG1, and IgG2a were added to the wells in triplicate, and incubation continued for 1 h at 37 °C. After washing, tetramethyl benzidine (TMB) was added as a substrate, and the absorbance was measured at 450 nm in a microplate reader Model 680 (Bio-Rad).

#### 2.8.4. Quantitative Real-Time PCR (qRT-PCR) 

The primers were designed and synthesized according to the GenBank cDNA sequences of mouse β-actin and the cytokines listed in Table 2. The pooled blood samples of each group (about 100 μL) were mixed with 1 mL of RNAiso plus (Takara, Dalian, China), and the total RNA was extracted using a Quantscript RT kit according to the manufacturer’s directions (TianGen Biotech, Beijing, China). Reverse-transcription PCR was performed using Transcript One-Step gDNA Removal and cDNA synthesis SuperMix (Transgen, Beijing, China). Real-time PCR was performed using a Bio-Rad iQ5 with 7 μL of cDNA, 7.5 μL of SsoFast EvaGreen SuperMix (Bio-Rad), and 0.25 μL of each of the forward and reverse primers in a total reaction volume of 15 μL.

A standard qRT–PCR protocol was carried out with an initial denaturation for 3 min at 95 °C, followed by 40 cycles of 10 s at 95 °C, and 20 s at 60 °C. Each run included a negative control with no template added. Beta-actin was used as a reference gene, and the mRNA levels of the immune-related genes were calculated by the geometric means method and the following formula: relative level = 2^−ΔΔCT^.

### 2.9. Statistical Analysis

The data from all of the groups are presented as means ± SD from the triplicate assays. Statistical analyses were determined by the statistical software program GraphPad Prism 7.0. The differences between the groups were analyzed by one-way ANOVA and Tukey’s test for multiple comparisons. The values of *p* < 0.05 were considered statistically significant, and vice versa. 

## 3. Results

### 3.1. Identification of Recombinant Plasmid

As shown in Supplementary Materials Figure S1, the bands of ~300 bp and ~1000 bp were detected by agarose gel electrophoresis following plasmid-PCR (Figure S1B(a,c)) and the double digestion (Figure S1B(b,d)) of the recombinant yeast, indicating that FBC and fused PIL-2/FBC had been successfully inserted into the pGAPZαA vector. Sequencing also confirmed this result (data not shown). 

### 3.2. Identification of Pichia Pastoris Recombinants

As shown in Supplementary Materials Figure S1, the bands of ~300 bp and ~1000 bp were detected by agarose gel electrophoresis following the RT-PCR of the recombinant yeast, indicating that FBC and fused PIL-2/FBC had been successfully integrated into the genome of Pichia pastoris. Sequencing also confirmed this result (data shown in the Supplementary Materials Figure S1C).

### 3.3. Bioactivity of Pichia Pastoris Recombinants In Vitro

**Figure 1:**
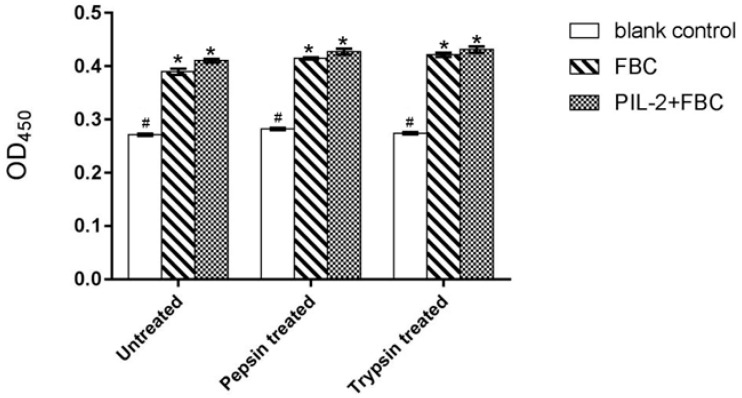
Proliferation of pig lymphoblasts treated by different Pichia pastoris recombinant supernatants. Blank control: pGAPZαA group. FBC: pGAPZαA-FBC group. PIL-2/FBC: pGAPZαA--PIL2/FBC. Untreated: the original recombinant yeast ferment supernatant; Pepsin treated: the recombinant yeast ferment supernatant digested with pepsin. Trypsin treated: the recombinant yeast ferment supernatant digested with trypsin. (*) *p* < 0.05 vs. blank control. (#) *p* < 0.05 vs. FBC. N = 3 (mean ± SD). The followings are all the same as here.

The CCK8 assay demonstrated that the proliferation of pig lymphoblasts in the experimental group (fused PIL-2/FBC) was significantly increased compared with the blank control group (empty plasmid) (*p* < 0.05). The difference between the PIL-2/FBC group and the FBC control group (just FBC, without pig IL-2) was not significant (*p* > 0.05), and their bioactivity was not markedly affected by enzyme digestion (Figure 1).

### 3.4. In Vitro Antimicrobial Activity of Pichia Pastoris Recombinants 

**Figure 2:**
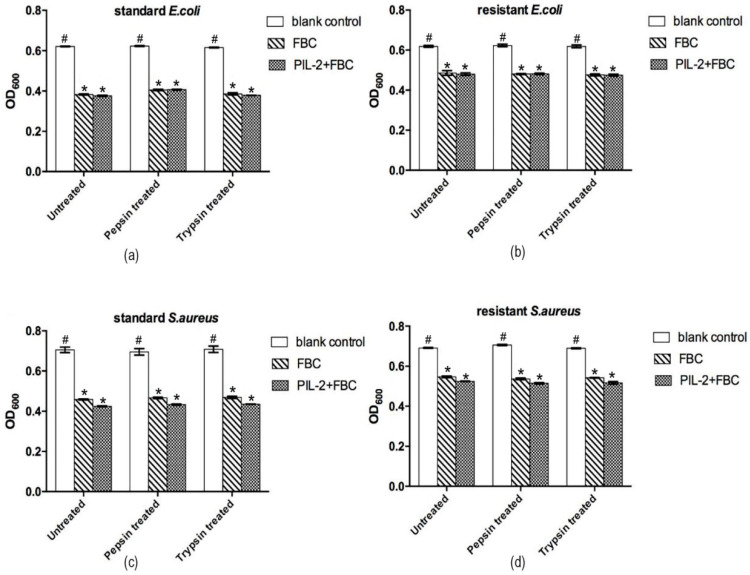
Antimicrobial activities of different recombinant Pichia pastoris supernatants in vitro. The bioactivity of the PIL-2/FBC co-expression protein was measured by inhibition of 4 pathogen strains, including standard *E. coli* ATCC25922 (**a**), *S. aureus* ATCC26112 (**c**); multi-antibiotic-resistant strains *E. coli* (**b**) and *S. aureus* (**d**). Untreated: the original recombinant yeast ferment supernatant; Pepsin treated: the recombinant yeast ferment supernatant digested with pepsin. Trypsin treated: the recombinant yeast ferment supernatant digested with trypsin. Blank control: pGAPZαA group. FBC: pGAPZαA-FBC group. PIL-2/FBC: pGAPZαA--PIL2/FBC. (*) *p* < 0.05 vs. blank control. (#) *p* < 0.05 vs. FBC. N = 3 (mean ± SD).

Compared to the blank control group, the supernatant of the PIL-2/FBC group and the FBC control group significantly inhibited the proliferation of all of the four tested bacteria (*p* < 0.05), but there was no significant difference between these two groups (*p* > 0.05) (Figure 2). The same results also appeared in the recombinant yeast ferment supernatants digested with pepsin or trypsin.

### 3.5. Body Weight

**Figure 3:**
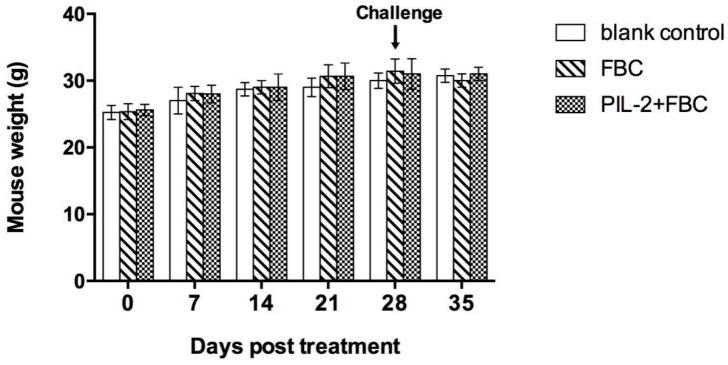
Mice weight change over the experimental period. Blank control: pGAPZαA group. FBC: pGAPZαA-FBC group. PIL-2/FBC: pGAPZαA—PIL2/FBC. N = 10 (mean ± SD).

Compared with the blank control group, the body weights of the PIL-2/FBC group and the FBC group mice were not significantly different over the entire observation period (*p* > 0.05) (Figure 3).

### 3.6. Leukocytes in Peripheral Blood

**Figure 4:**
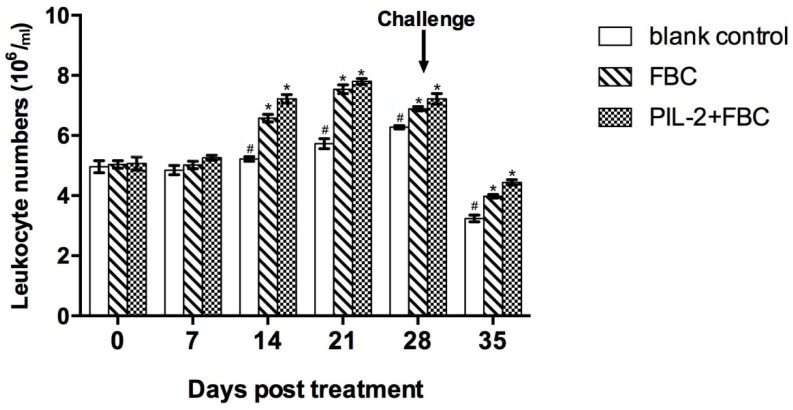
Changes of leukocytes in the peripheral blood of experimental mice. The proliferation of treated groups PL-2+FBC and FBC had significant proliferation compared with the control group from 14 to 35 days (*p* < 0.05), and the proliferation of PIL-2+FBC was not significantly different from FBC over the entire experimental period. Blank control: pGAPZαA group. FBC: pGAPZαA-FBC group. PIL-2/FBC: pGAPZαA--PIL2/FBC. (*) *p* < 0.05 vs. blank control. (#) *p* < 0.05 vs. FBC. N = 10 (mean ± SD).

Compared with the blank control group, the leukocytes of the PIL-2/FBC group and the FBC group increased significantly from 14 to 35 days post-treatment (*p* < 0.05), and the difference between these two groups was not significant from 7 to 35 days (*p* > 0.05) (Figure 4).

### 3.7. CD4+ and CD8+ T Cells Numbers

**Figure 5:**
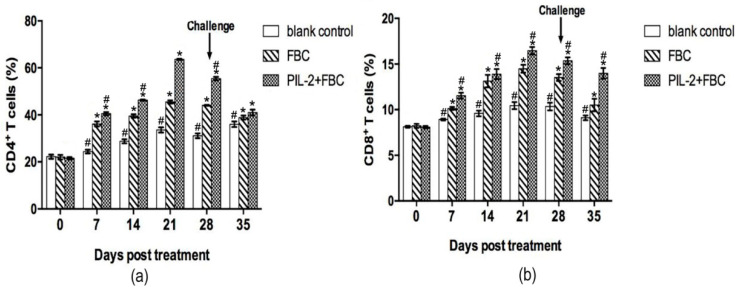
Changes of CD4^+^ (**a**) and CD8^+^ T (**b**) cells in the peripheral blood of mice. The amounts of CD4^+^ and CD8^+^ T cells significantly increased in the blood of treated groups PIL-2+FBC and FBC from 7 to 35 days compared with the control group (*p* < 0.05), moreover, the amounts of CD4^+^ and CD8^+^ T cells in PL-2+FBC group were higher than FBC from 7 to 35 days (P<0.05). Blank control: pGAPZαA group. FBC: pGAPZαA-FBC group. PIL-2/FBC: pGAPZαA--PIL2/FBC. (*) *p* < 0.05 vs. blank control. (#) *p* < 0.05 vs. FBC. N = 10 (mean ± SD).

After treatment, the CD4^+^ and CD8^+^ T cells markedly increased from 7 days onwards in the PIL-2/FBC group and the FBC group compared with the blank control group (*p* < 0.05) (Figure 5), and the differences were also significant between the PIL-2/FBC group and the FBC group from 7 to 35 days (*p* < 0.05).

### 3.8. Levels of IgG

**Figure 6:**
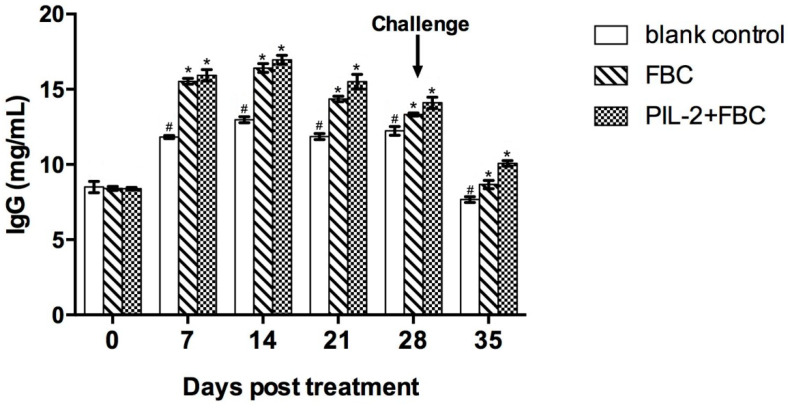
Change of IgG in the sera of the experimental mice by ELISA. The IgG level of treated group PL-2+FBC and FBC were higher than that of the control group from day 7 to day 35 post-treatment (*p* < 0.05). There was no difference between PL-2+FBC group and FBC group (*p* < 0.05). Blank control: pGAPZαA group. FBC: pGAPZαA-FBC group. PIL-2/FBC: pGAPZαA--PIL2/FBC. (*) *p* < 0.05 vs. blank control. (#) *p* < 0.05 vs. FBC. N = 10 (mean ± SD).

Compared with the blank control group, IgG significantly increased in the sera of the experimental group and the FBC control group from 7 days thereafter (*p* < 0.05), but the difference in IgG was not significant between the two treated groups (*p* > 0.05) (Figure 6).

### 3.9. Immune Gene Expression

#### 3.9.1. Toll-Like Receptor Gene

**Figure 7:**
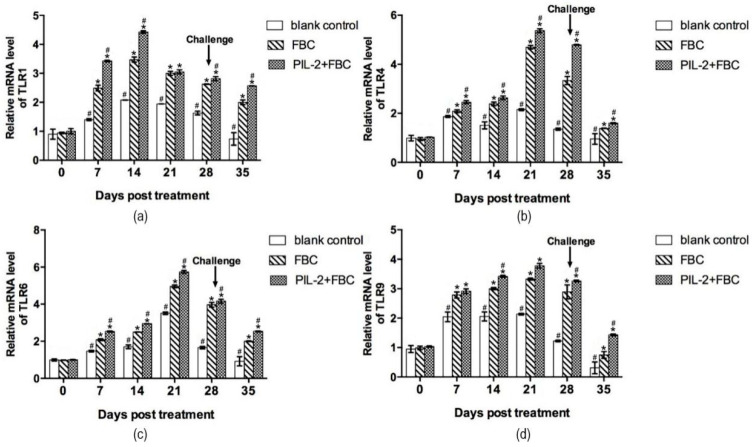
Expression of mRNA levels of TLR-1 (**a**), TLR-4 (**b**), TLR-6 (**c**), and TLR-9 (**d**) gene in the blood of experimental mice. The expression of TLR-1, TLR-4, TLR-6 and TLR-9 increased markedly in PL-2+FBC group and FBC group on different days post-treatment compared with the control group (*p* < 0.05), and the expression of these four TLR genes of PL-2+FBC were promoted significantly in different periods after treatment compared with FBC (*p* < 0.05). Blank control: pGAPZαA group. FBC: pGAPZαA-FBC group. PIL-2/FBC: pGAPZαA--PIL2/FBC. (*) *p* < 0.05 vs. blank control. (#) *p* < 0.05 vs. FBC. N = 10 (mean ± SD).

Figure 7 shows that the fused PIL-2/FBC group manifested the highest increases in TLR-1, TLR-4, TLR-6, and TLR-9 mRNA expression compared to the two control groups (*p* < 0.05), and after the challenge, the mRNA expressions of TLRs in the PIL-2/FBC group were also significantly higher than the FBC and blank control mice (*p* < 0.05).

#### 3.9.2. Immune Memory-Related Genes

**Figure 8:**
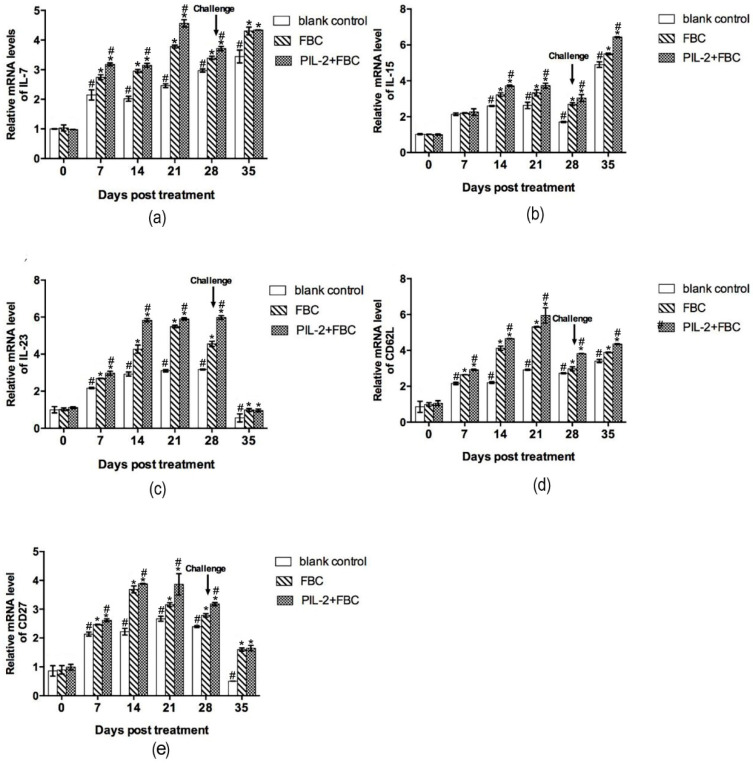
Expression of mRNA levels of IL-7 (**a**), IL-15 (**b**), IL-23 (**c**), CD62L (**d**), and CD27 (**e**) gene in the blood of experimental mice. The expression levels of IL-7, IL-15, IL-23, CD62L, and CD27 genes in treated groups had significant increases compared with the control group from day 14 to 35 (*p* < 0.05). The group treated with PIL-2/FBC achieved a higher expression level of these five genes at 7 to 28 days in comparison with FBC, except IL-15 on day 7 (*p* < 0.05). Blank control: pGAPZαA group. FBC: pGAPZαA-FBC group. PIL-2/FBC: pGAPZαA—PIL2/FBC. (*) *p* < 0.05 vs. blank control. (#) *p* < 0.05 vs. FBC. N = 10 (mean ± SD).

Figure 8 shows the changes in the expressions of immune memory-related genes IL-7, IL-15, IL-23, CD62L, and CD27. From day 14 to 21, the expression levels of all five genes in the PIL-2/FBC and FBC group significantly increased compared to the blank control group (*p* < 0.05), and the levels of the PIL-2/FBC treated mice were significantly higher than those of the FBC group from day 7 to day 28, except for IL-15 on day 7 (*p* < 0.05).

#### 3.9.3. Cytokine Genes 

**Figure 9:**
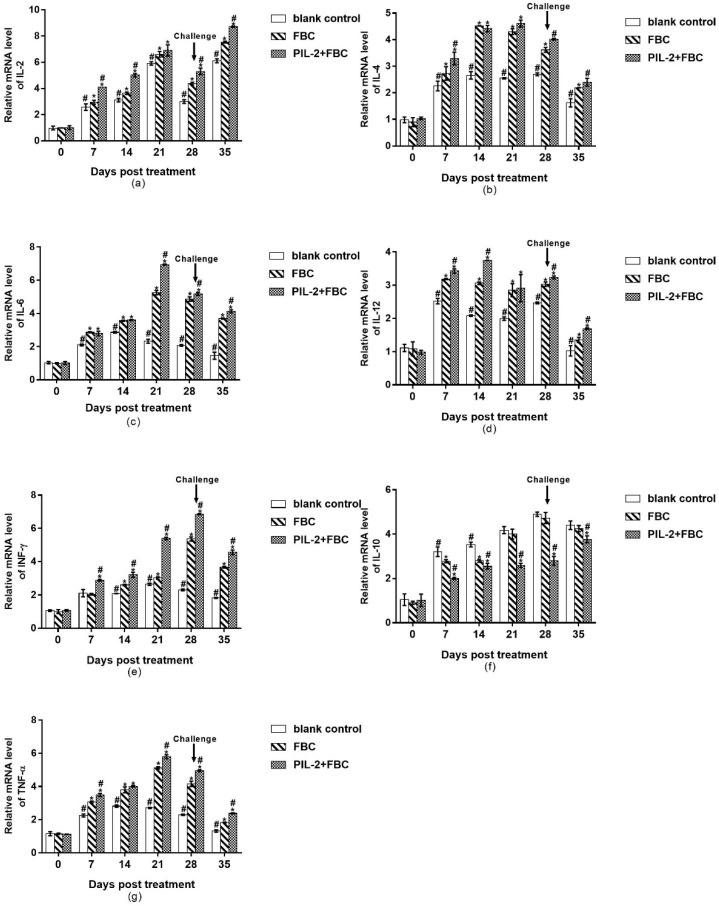
Expression of mRNA levels of IL-2 (**a**), IL-4 (**b**), IL-6 (**c**), IL-12 (**d**), IFN-γ (**e**), IL-10 (**f**), and TNF-α (**g**) genes in the blood of the experimental mice. All the gene expression levels of treated groups were significantly increased compared with the control group except IL-10 (*p* < 0.05), and the IL-4, IFN-γ and TNF-α of PIL-2/FBC group elevated significantly compared to FBC group from 28 to 35 days or 21 to 35 days, respectively, (*p* < 0.05); While IL-10 of PIL-2/FBC group remarkably decreased compared with the control group and FBC group during the whole experiment period (*p* < 0.05). Blank control: pGAPZαA group. FBC: pGAPZαA-FBC group. PIL-2/FBC: pGAPZαA--PIL2/FBC. (*) *p* < 0.05 vs. blank control. (#) *p* < 0.05 vs. FBC. N = 10 (mean ± SD).

The expression levels of IL-2, IL-4, IL-6, IL-12, IFN-γ, and TNF-α were all remarkably higher in the PIL-2/FBC group and the FBC control group than those of the blank control group from 7 to 35 days (Figure 9a–f), and the IL-4, IFN-γ, and TNF-α of the PIL-2/FBC group elevated significantly compared to the FBC group from 28 to 35 days or 21 to 35 days, respectively, (*p* < 0.05); while the IL-10 of the PIL-2/FBC group remarkably decreased compared with the two control groups during the whole experiment period (*p* < 0.05) (Figure 9f).

#### 3.9.4. Antimicrobial Peptides

**Figure 10:**
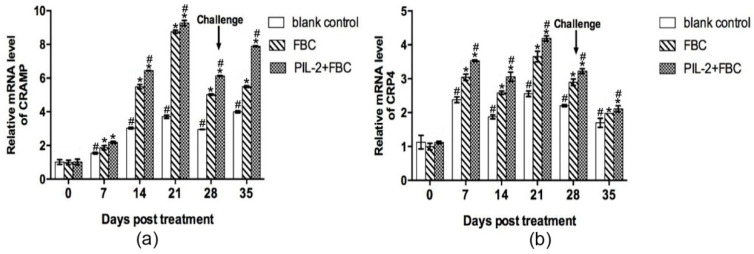
Expression of mRNA levels of CRAMP (**a**) and CRP4 (**b**) gene in the blood of the experimental mice. It was remarkable that PIL-2/FBC and FBC group achieved significant increases from day 7 to 35 compared to the control group (*p* < 0.05). In addition, the expression of CRAMP and CRP4 in PIL-2+FBC group was higher than that of FBC group. Blank control: pGAPZαA group. FBC: pGAPZαA-FBC group. PIL-2/FBC: pGAPZαA--PIL2/FBC. (*) *p* < 0.05 vs. blank control. (#) *p* < 0.05 vs. FBC. N = 10 (mean ± SD).

Figure 10 shows that in comparison with the blank control group, the levels of the CRAMP and CRP4 antimicrobial peptides increased significantly in PIL-2/FBC and the FBC group from day 7 to 35 (*p* < 0.05), while the level of CRAMP in the FBC group was lower than the PIL-2/FBC t group from day 14 to 35 (*p* < 0.05).

### 3.10. Virulent Challenge

**Figure 11:**
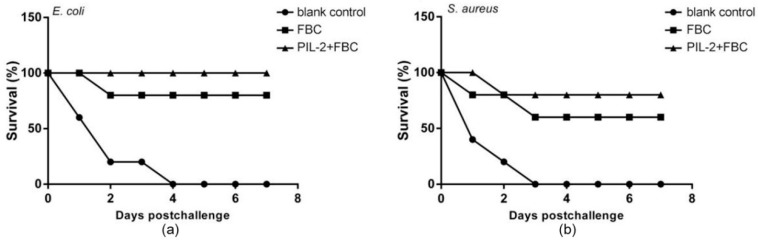
Survival of mice challenged with virulent *E. coli* (**a**) and *S. aureus* (**b**). It was notable that the mice treated with PIL-2+FBC, respectively, showed 100% and 80% protection rates against *E. coli* (**a**) and *S. aureus* (**b**). Moreover, the group treated with FBC showed a protection rate of 80% and 60% against *E. coli* and *S. aureus*, respectively. In comparison, the control group died within 3 days post-challenge. Blank control: pGAPZαA group. FBC: pGAPZαA-FBC group. PIL-2/FBC: pGAPZαA--PIL2/FBC, ten mice per group.

At 28 days post-treatment, the mice were, respectively, challenged with the intraperitoneal injection of virulent bacteria *E. coli* and *S. aureus*, and the PIL-2/FBC and FBC group showed 100% and 80% survival rates against *E. coli* infection compared with 100% mortality in the blank control group (Figure 11a). While the majority of the FBC group (60%) and PIL-2/FBC group (80%) survived *S. aureus* infection, and all mice of the blank control group died within 3 days post-challenge (Figure 11b). In addition, visual examination revealed no pathological lesions in the organs of the surviving mice. In contrast, the control mice died from infection with overt lesions, including edema of the liver and spleen, diffuse bleeding from the stomach, duodenum, and jejunal catarrh.

## 4. Discussion

With the rampant increase and expansion of drug-resistant bacteria, the development of anti-infection immunopotentiators, such as cytokines and antibacterial peptides, represents a new, safe, and effective coping strategy in this era of emerging superbugs [20,21]. Immunopotentiators could avoid the rapid evolution of microbial resistance under selective pressure because of targeting the host rather than the pathogen. In recent years, immunopotentiators are usually employed as a potent therapy to correct acquired or innate defects of the immune response; and in most cases, various pathogenic infections often suppress the normal immune response via secreting specific virulent inhibitors. For example, IL-2 has been used to treat metastatic cancer [22] and multi-drug-resistant tuberculosis [23], and shuffled IL-2 has also been shown to be an effective adjuvant to boost the immune response to the pasteurella multocida vaccine [24]. IDR1018 contributed to the innate immune response to protect the host against microbial infections through the modulation of neutrophil function [25]. However, most of the studies on anti-infection immunopotentiators have focused on the effects of one sided immune response, either innate or adaptive immunity. In fact, effective protection often comes from the orchestrated collaboration of innate and adaptive immunity through the regulation of cytokines.

Pig breeding is the most important husbandry in China, and it is still susceptible to various infections that cause huge economic losses to pork production. In addition to the traditional biosafety control measures, antibiotics and vaccines are not enough for the prevention of infectious diseases in massive-scale pig farms. Therefore, we attempted to develop a novel and effective immunopotentiator to enhance the resistance of pigs against various infectious pathogens. Interleukins are vital regulatory cytokines to control the innate and adaptive immunity of pig against pathogens. Based on our previous experience, pig IL-2 is especially suitable for promoting the adaptive immunity of pigs against pathogens [13]. In fact, IL-2 can safely increase the immunity of animals without obvious side effects at appropriate doses in vivo. Therefore, we consider IL-2 to be safer and more powerful than other cytokines in increasing the cellular immunity of animals against pathogens according to immunological theory and our past trials. Moreover, the bioactivity of IL-2 does not conflict or overlap with bovine cathelicidin, and in theory, it could motivate immune cells, such as macrophages, NK and DC, to produce more bioactive molecules and result in better phagocytosis and stronger eradication of pathogens in animals. 

Previous studies have shown that the four cationic host defense peptides that comprise FBC played important roles in innate immunity, not only directly killing pathogens but also promoting immunomodulation through the induction of cytokines. We speculated that bovine cathelicidin could overcome the increasing antibiotic resistance occurring in pig farms in China, which rarely encounter virulent porcine pathogens. Hence, we selected the shuffled pig IL-2 and bovine cathelicidins to be co-expressed in yeast vectors to develop a new, economical, and effective immunomodulator. 

Herein pig IL-2 and FBC genes were firstly co-expressed using the self-cleavage 2A peptide technique, which permits multiple genes to be equally expressed in the same vector [26,27]. Our results showed that recombinant IL-2 and FBC significantly promoted the proliferation of pig lymphoblast in vitro and increased the number of leukocytes, CD4+ and CD8+ T cells, IgG, and the gene expressions of TLRs (TLR1,4,6,9), antimicrobial peptides (CRP4 and CRAMP) and interleukins (IL-2, 4, 6, 7, 12, 15, and 23), IFN-γ and TNF-α in the blood in vivo both before and after challenge. The enhancement of these immunities (competent immune cells, IgG, TLRs, and cytokines) may be attributed to expressed IL-2 that, as a master regulator mediating adaptive immunity, can not only induce T-cell proliferation and maturation but also modulate CD4+ T cells into Th1 cells along with naïve CD8+ T cells into either effector or memory T cells [28]. Th1 cells are known to mediate host defense against pathogens via secreting IFN-γ and IL-2 [29], and CD8+ T effector cells could defeat infection by the production of IFN-γ, perforin, and granzymes [30].

Obviously, the elevated expression of TLRs probably resulted from the combined regulation of IL-2 and FBC, and the fused bovine cathelicidins contain IDR1002, indolicidin, Bac2A, and CP10A. As previous studies have shown that the four cationic host defense peptides played important roles in innate immunity, not only directly killing pathogens but also promoting immunomodulation through the induction of chemokines [8,9,10,11]. Notably, our results suggested that the contents of the interleukins in the fused PIL-2/FBC-treated group, except for IL-10, significantly increased in comparison with the control groups in different periods. Furthermore, the PIL-2/FBC mice displayed better protection with higher survival levels after the challenge. This could possibly be explained from two aspects: one is the collaboration of co-expressed IL2 and FBC to promote both innate and adaptive immune function effectively; another is the decrease in IL-10 that leads to the better clearance of pathogens because IL-10 is a suppressive cytokine. Previous studies have shown that IL-10 could inhibit the immune response to pathogens that cause chronic infection and prevent damaging inflammation [31,32]. Many cells, including dendritic cells, macrophages, and Th1 and Th2 cells, could secrete IL-10 [32,33]. The decrease in IL-10 probably helps raise immune functions due to the co-expression of IL-2 and FBC. Meanwhile, IL-2 can induce CD4+ T cells to differentiate into Th1 cells to down-regulate IL-10 production. Similarly, IDR1002 fused into FBC was known to reduce the production of IL-10 [25].

Compared to intramuscular injection, oral inoculation is generally considered to be the feasible treatment with several advantages, including lack of invasiveness, safety, simplicity, and lower costs. However, oral inoculation frequently resulted in poor efficacy due to the harsh environment of the gastrointestinal tract [34]. In order to avoid enzymatic digestion in the gut and to improve the expression in vivo, IL2 and FBC were expressed in Pichia pastoris, which is an effective encapsulating vehicle and a safe, commonly used vector for the expression of heterologous genes for oral use [15,35,36]. The results of simulating enzyme digestion in the digestive tract showed that the lymphocyte proliferation activity of IL-2 and the antimicrobial efficacy of FBC were not severely affected by pepsin and trypsin in vitro. The fused PIL-2/FBC-treated mice did not show any obvious lesion or systemic symptom after challenge with virulent drug-resistant *E. coli* and *S. aureus*. Taken together, these also confirmed that Pichia pastoris is an effective and safe delivery for co-expressed IL-2 and FBC immunopotentiator. 

By the way, we just used the Pichia pastoris strain SMD1168 with empty plasmid pGAPZαG as the negative control, which was not enough to elucidate the contribution of blank yeast alone. Moreover, we just performed RT-PCR for IL-2 and FBC in vitro but failed to detect FBC in the in vivo experiment. To some extent, our present data just proved the expression of IL2 and FBC in vitro and their respective bioactivity in animal experiments. Their molecular mechanisms for systematic immune regulation should be solved in the forthcoming experiment. 

## 5. Conclusions

In summary, our results suggest that the co-expressed pig IL-2 and FBC can provide potent antibacterial defense against microbial infection, which can be developed into a safe and effective immunopotentiator for the prevention of infectious diseases in the antibiotic-resistance era. The detailed underlying mechanism for the immune enhancement of co-expressed pig IL-2 and FBC remains unclear, which could be further explored in subsequent experiments.

## Figures and Tables

**Table 1:** The primers for pig IL-2, 2A-α-factor and FBC.

Primer	Oligonucleotide Sequences (5′-3′)
IL-2-F	GAAGCTGAATTCATGTATAAGATGCAGCTCTTGT
IL-2-R	CGCATGTTAGAAGACTTCCCCTGCCCTCTCCGCTTCCAGTCAGTGTTGAGTAGATGCT
2A-α-F	CTTCTAACATGCGGGGACGTGGAGGAAAATCCCGGGCCAATGAGATTTCCTTCAATTTTTAC
2A-α-R	TGCCAATCGAGCTTCAGCCTCTCTTTTCT
FBC-F	GAGGCTGAAGCTCGATTGGCACGTATCGTCGT
FBC-R	CGGTGTTCTAGACTAATGGTGATGGTGATGAT
FBC-F’	GAAGCTGAATTCCGATTGGCACGTATCGTCGT
FBC-R’	AAACGGGCCCTCTAGACTAATGGTGATGGTGATGATGCT

F: forward. R: reverse, GAATTC (EcoRI restriction site); TCTAGA (Xbal restriction site).

**Table 2:** The primers for RT-qPCR.

Gene	Oligonucleotide Sequences (5′-3′)
β-actin-F	TACGCCAACACGGTGCTGTC
β-actin-R	GTACTCCTGCTTGCTGATCCACAT
TLR-1-F	GGACCTACCCTTGCAAACAA
TLR-1-R	GGTGGCACAAGATCACCTTT
TLR-4-F	ACCTGGCTGGTTTACACGTC
TLR-4-R	CTGCCAGAGACATTGCAGAA
TLR-6-F	CCAAGAACAAAAGCCCTGAG
TLR-6-R	TGTTTTGCAACCGATTGTGT
TLR-9-F	ACTGAGCACCCCTGCTTCTA
TLR-9-R	AGATTAGTCAGCGGCAGGAA
IL-2-F	AAGCACAGCAGCAGCAGCAG
IL-2-R	GCCGCAGAGGTCCAAGTTCATC
IL-4-F	GCCATATCCACGGATGCGACAA
IL-4-R	GGTGTTCTTCGTTGCTGTGAGGA
IL-6-F	TCTTGGGACTGATGCTGGTGACA
IL-6-R	AGCCTCCGACTTGTGAAGTGGTAT
IL-7-F	TTCCTCCACTGATCCTTGTTCT
IL-7-R	AGCAGCTTCCTTTGTATCATCAC
IL-10-F	GCTCTTACTGACTGGCATGAG
IL-10-R	CGCAGCTCTAGGAGCATGTG
IL-12-F	CAATCACGCTACCTCCTCTTTT
IL-12-R	CAGCAGTGCAGGAATAATGTTTC
IL-15-F	CATCCATCTCGTGCTACTTGTG
IL-15-R	GCCTCTGTTTTAGGGAGACCT
IL-23-F	TGCTGGATTGCAGAGCAGTAA
IL-23-R	GCATGCAGAGATTCCGAGAGA
IFN-γ-F	AGGCCATCAGCAACAACATA
IFN-γ-R	TGAGCTCATTGAATGCTTGG
TNF-α-F	CCTGTAGCCCACGTCGTAG
TNF-α-R	GGGAGTAGACAAGGTACAACCC
CRP4-F	AGAGGTTTGTTATGCTATTGTAGA
CRP4-R	TCAGCGGCGGGGGCAG
CAMP-F	CAGCAGTCCCTAGACACCAAT
CAMP-R	CACAGACTTGGGAGTATCTGGA
CD62L-F	TACATTGCCCAAAAGCCCTTAT
CD62L-R	CATCGTTCCATTTCCCAGAGTC
CD27-F	CAGCTTCCCAACTCGACTGTC
CD27-R	GCACCCAGGACGAAGATAAGAA

F: forward. R: reverse.

## Data Availability

The Supplementary Material for this article can be found at Biology website, https://www.mdpi.com/article/10.3390/biology11101491/s1. The original contributions presented in the study are included in the article/Supplementary Material. Further inquiries can be directed to the corresponding author.

## Supplementary Materials

The following supporting information can be downloaded at: https://www.mdpi.com/article/10.3390/biology11101491/s1, Figure S1: The flow diagram of recombinant plasmid pGAPZαA-PIL2-FBC construction (A), electrophoretic analysis of *Pichia pastoris* recombinants (1% agarose gel) (B) and RT-PCR of Pichia pastoris recombinants (1% agarose gel) (C). (Ba) Lane M: 20bp DNA ladder marker; Lane 1: Electrophoresis of the PCR fragment of pGAPZαA-FBC. Lane 2: PCR product of pGAPZαA plasmid. (Bb) Lane M: 20bp DNA ladder marker; Lane 1: Electrophoretic identification of double digested pGAPZαA-FBC/EcoRI+Xbal. Lane 2: pGAPZαA plasmid. (Bc) Lane M: Trans 2K plus DNA ladder marker; Lane 1: Electrophoresis of the PCR fragment of pGAPZαA-PIL-2/FBC. Lane 2: PCR product of pGAPZαA plasmid. (Bd) Lane M: Trans 2K plus DNA ladder marker; Lane 1: Electrophoretic identification of double digested pGAPZαA-PIL-2/FBC /EcoRI+Xbal. Lane 2: pGAPZαA plasmid. (Ca) Lane M: 20bp DNA ladder marker; Lane 1: Electrophoresis of the RT-PCR fragment of SMD-pGAPZαA-B (FBC). Lane 2: RT-PCR fragment of SMD-pGAPZαA. (Cb) Lane M: Trans 2K plus DNA ladder marker; Lane 1: Electrophoresis of the RT-PCR fragment of SMD-pGAPZαA-IL2-B (PIL-2/FBC). Lane 2: RT-PCR fragment of SMD-pGAPZαA.
