# Supplementary material for: Co-Expression of Pig IL-2 and Fusion Bovine Cathelicidin Gene by Recombinant Plasmids in Yeast and Their Promotion of Mouse Antibacterial Defense
Source: Biology (Basel). 2022 Oct 12;11(10):1491. doi: 10.3390/biology11101491 (PMC9598770; doi:10.3390/biology11101491)
Supplement: Supplementary file 1 [file biology-11-01491-s001.zip › biology-1918947-supplementary.pdf]

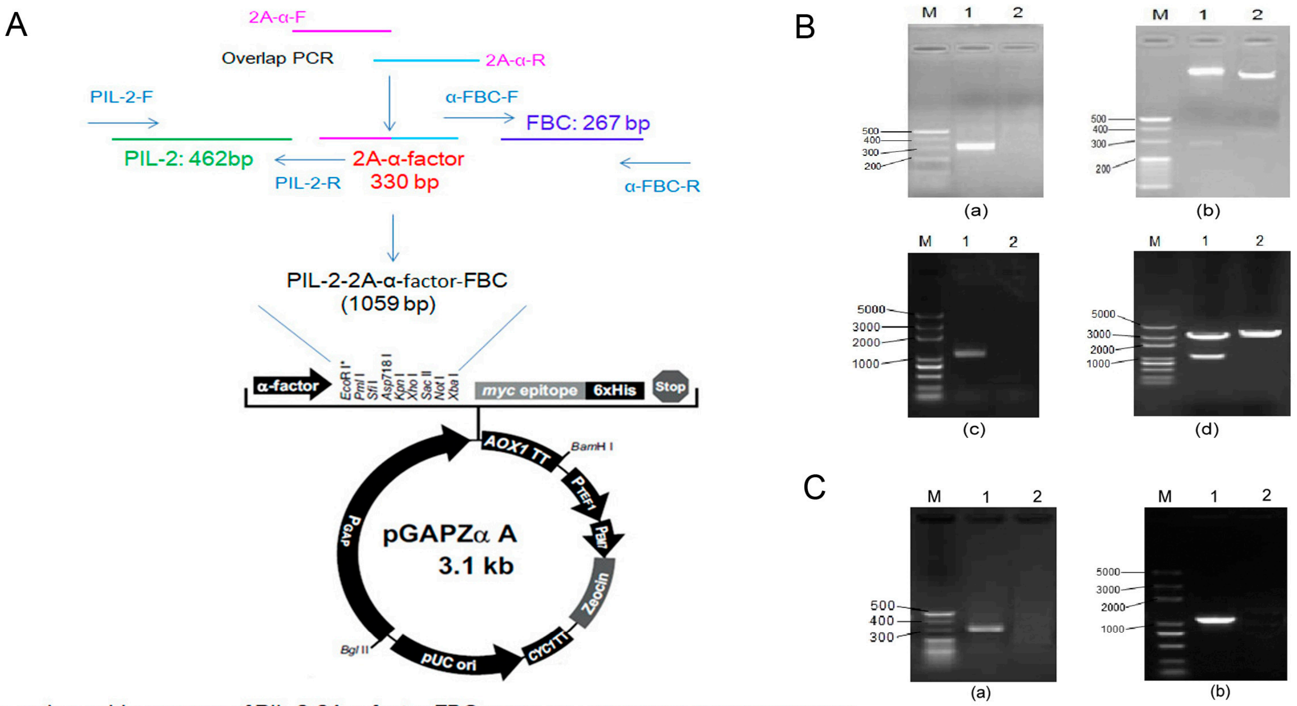

The amino-acid sequence of PIL-2-2A-α-factor-FBC : MYKMQLLCCIALTLALMANGAPTSSSTKNT
KKQLEPLLLDLQLLLKEVKNYENADLSRMLTFKFYMPKQATELKHLQCLVEELKALEGVLNLGQSKNSDSANIKES
MNNINVTVLELKGSETSFKCEYDDETVTAVEFLNKWITFCQSIYSTLTGSGEGRGSLLTCGDVEENPGPMRFPSIFT
AVLFAASSALAAPVNTTTEDETAQIPAEAVIGYSDLEGDFDVAVLPFSNSTNNGLLFINTTIASIAAKEEGVSLEKREA
EARLARIVVIRVARGSGDDDDKVQRWLIVWRIRKGSGDDDDKILPWKWPWWPWRRGGSGDDDDKILAWKW
AWWAWRRGSGDDDDKHHHHHH

supplementary materials:

Figure S1. The flow diagram of recombinant plasmid pGAPZαA-PIL2-FBC construction (A), electrophoretic analysis of *Pichia pastoris* recombinants (1% agarose gel) (B) and RT-PCR of Pichia pastoris recombinants (1% agarose gel)(C). (Ba) Lane M: 20bp DNA ladder marker; Lane 1: Electrophoresis of the PCR fragment of pGAPZαA-FBC. Lane 2: PCR product of pGAPZαA plasmid. (Bb) Lane M: 20bp DNA ladder marker; Lane 1: Electrophoretic identification of double digested pGAPZαA-FBC/EcoRI+XbaI. Lane 2: pGAPZαA plasmid. (Bc) Lane M: Trans 2K plus DNA ladder marker; Lane 1: Electrophoresis of the PCR fragment of pGAPZαA- PIL-2/FBC. Lane 2: PCR product of pGAPZαA plasmid. (Bd) Lane M: Trans 2K plus DNA ladder marker; Lane 1: Electrophoretic identification of double digested pGAPZαA- PIL-2/FBC /EcoRI+XbaI. Lane 2: pGAPZαA plasmid. (Ca) Lane M: 20bp DNA ladder marker; Lane 1: Electrophoresis of the RT-PCR fragment of SMD-pGAPZαA-B (FBC). Lane 2: RT-PCR fragment of SMD-pGAPZαA. (Cb) Lane M: Trans 2K plus DNA ladder marker; Lane 1: Electrophoresis of the RT-PCR fragment of SMD-pGAPZαA-IL2-B (PIL-2/FBC). Lane 2: RT-PCR fragment of SMD-pGAPZαA.
